# Seizure Onset Zone Lateralization Using a Non-linear Analysis of Micro vs. Macro Electroencephalographic Recordings During Seizure-Free Stages of the Sleep-Wake Cycle From Epilepsy Patients

**DOI:** 10.3389/fneur.2020.553885

**Published:** 2020-09-17

**Authors:** Cristina G. B. Martínez, Johannes Niediek, Florian Mormann, Ralph G. Andrzejak

**Affiliations:** ^1^Department of Communication and Information Technologies, Universitat Pompeu Fabra, Barcelona, Spain; ^2^Edmond and Lily Safra Center for Brain Sciences, The Hebrew University of Jerusalem, Jerusalem, Israel; ^3^Department of Epileptology, University of Bonn, Bonn, Germany

**Keywords:** epilepsy, quantitative EEG analysis, seizure onset zone lateralization, EEG, hybrid electrodes

## Abstract

The application of non-linear signal analysis techniques to biomedical data is key to improve our knowledge about complex physiological and pathological processes. In particular, the use of non-linear techniques to study electroencephalographic (EEG) recordings can provide an advanced characterization of brain dynamics. In epilepsy these dynamics are altered at different spatial scales of neuronal organization. We therefore apply non-linear signal analysis to EEG recordings from epilepsy patients derived with intracranial hybrid electrodes, which are composed of classical macro contacts and micro wires. Thereby, these electrodes record EEG at two different spatial scales. Our aim is to test the degree to which the analysis of the EEG recorded at these different scales allows us to characterize the neuronal dynamics affected by epilepsy. For this purpose, we retrospectively analyzed long-term recordings performed during five nights in three patients during which no seizures took place. As a benchmark we used the accuracy with which this analysis allows determining the hemisphere that contains the seizure onset zone, which is the brain area where clinical seizures originate. We applied the surrogate-corrected non-linear predictability score (ψ), a non-linear signal analysis technique which was shown previously to be useful for the lateralization of the seizure onset zone from classical intracranial EEG macro contact recordings. Higher values of ψ were found predominantly for signals recorded from the hemisphere containing the seizure onset zone as compared to signals recorded from the opposite hemisphere. These differences were found not only for the EEG signals recorded with macro contacts, but also for those recorded with micro wires. In conclusion, the information obtained from the analysis of classical macro EEG contacts can be complemented by the one of micro wire EEG recordings. This combined approach may therefore help to further improve the degree to which quantitative EEG analysis can contribute to the diagnostics in epilepsy patients.

## 1. Introduction

Epilepsy is among the most common serious neurobiological disorders worldwide affecting almost 1% of the world's population (1, 2). In roughly two thirds of these patients, complete seizure control can be achieved by anti-epileptic medication. However, the remaining patients do not become seizure-free on adequate drug therapy or side effects of this treatment are not well-tolerated (3). Around 25–50% of these pharmacoresistant epilepsy patients suffer from focal epilepsy (4), meaning that initial seizure discharges can be recorded from a localized region in the brain, the so-called seizure onset zone (SOZ) (5). For these patients, the identification and neurosurgical removal of the SOZ (6) can be the only chance for cure. Non-invasive seizure monitoring along with structural imaging and other diagnostic pillars can lead to a correct lateralization of the SOZ in the majority of the cases. However, for ~10–20% of the patients invasive seizure monitoring using intracranially implanted electrodes can be indicated to reach a clinical decision about resectability (7, 8). The gold standard for SOZ localization is the visual inspection of electroencephalographic (EEG) recordings (9, 10). In recent years, this visual analysis of the EEG was progressively complemented by quantitative signal analysis techniques. These techniques assess EEG characteristics that are difficult or impossible to extract by visual inspection alone. Our particular type of analysis can therefore be useful in the SOZ lateralization of patients undergoing invasive seizure monitoring. Some of these techniques are used for the characterization of seizure activity, the so-called ictal activity (9–22), while others are used for the analysis of the seizure-free interval (23–62). The characterization of the seizure-free interval, often referred to as interictal interval, can reveal aspects of brain dynamics that may help in the localization of the SOZ without the need to wait for seizures to occur. Such analysis can therefore help to reduce the invasive monitoring time and minimize the patients' risk. Different approaches have been applied to seizure-free EEG recordings in order to localize the SOZ (23–34, 36–38, 40, 41, 43, 45–62) or to predict the surgical outcome for individual patients (35, 36, 39, 42, 44). Measures derived from linear signal analysis have been used for the characterization of the seizure-free interval of EEG recordings, such as spectral analysis (23–28, 55), linear cross correlation (29, 30, 34, 51), Pearson's cross correlation (34, 35), Spearman rank correlation coefficient (44), autocorrelation decay time (55), linear coherence (31, 32), genuine linear cross correlation (33), linear autoregressive models (63), or Granger Causality (36, 37, 40). In addition, non-linear signal analysis, such as non-linear correlation (41–43), Hilbert phase synchronization (29, 30, 34, 45, 46), event phase synchronization (47), mutual information (34), non-linear interdependence measures (30, 48–51), transfer entropy (52), symbolic transfer entropy (53), synchronization likelihood (62), non-linear measures of predictability (48, 54–56, 64), non-stationarity (48), correlation sum (57), neural mass models (65), non-linear autoregressive models (63), or effective correlation dimension (58–61, 64) have been applied. Furthermore, linear and non-linear signal analysis measures were combined with the concept of surrogates to analyze seizure-free EEG recordings (33, 48, 51, 54–57, 59). These studies provide converging evidence that characteristics of EEG recorded from the SOZ are different as compared to those recorded from outside of the SOZ. Accordingly, such an analysis can contribute to the localization of the SOZ.

Intracranial electrophysiological measurements in epilepsy patients can provide access to the activity of single neurons (5, 66–68). Currently, the so-called Utah arrays (45, 69–77), and micro-wires integrated into intracranial hybrid depth EEG electrodes can record neural activity at this micro-scale. These hybrid depth electrodes are equipped with conventional macro contacts and micro wires protruding along the electrode shaft or from the shaft tip (70, 78–97). Thereby, hybrid depth electrodes record electrical activity of the brain at two different spatial scales. Typically, micro-electrodes and micro-wires are used to study the low (45, 70, 72–75, 77–80) and the high-frequency component (76, 80–87) of the local field potentials (LFP), and single unit activity of neurons (69–72, 75, 77, 78, 80, 88–97). Despite the potential importance of quantitative analysis of EEG recordings performed at different spatial scales, the connection between macro- and micro- EEG recordings has not been widely investigated. Worrel et al. were the first to compare the suitability of macro-electrodes and micro-wires recording high frequency oscillations (HFOs) in interictal EEG recorded during slow-wave sleep with hybrid depth electrodes. The study concluded that HFOs were better recorded with micro-wires (81). Other studies showed that macro-electrodes provide only minimal advantages over micro-wires to record events in the low range of HFOs (86, 87), whereas micro-wires record HFOs of higher frequency than macro electrodes (87). Regarding seizure activity, different studies observed that it was detectable on individual micro electrodes (Utah arrays) (98, 99) and individual micro wires (hybrid depth electrodes) (79, 80) before it was observed in macro electrodes.

Andrzejak et al. applied different univariate (55) and bivariate (51) signal analysis measures to interictal EEG recordings from epilepsy patients and compared the accuracy of linear, non-linear, and surrogate corrected non-linear approaches with regard to the localization of the SOZ. Linear signal analysis measures, such as the autocorrelation function are most suited to extract characteristics of linear dynamics. However, they are not sensitive to certain properties of non-linear dynamics. Non-linear signal analysis techniques are needed to capture these non-linear properties. While non-linear techniques are sensitive to characteristics of non-linear dynamics, they usually lack specificity since they are also influenced by properties, such as the linear autocorrelation. This lack of specificity can be overcome by the concept of surrogates. Surrogates are generated from a constrained randomization of the original signals. Only certain properties of the original signals are preserved. Accordingly, surrogates are designed to test a specified null hypothesis about the dynamics underlying the signals. The surrogate corrected measures are thereby expected to be more specific for properties of non-linear dynamics. The results of the aforementioned studies (51, 55), in close agreement to other studies on interictal EEG periods (33, 48, 54, 56, 57, 59, 64), showed a substantial advantage of the surrogate corrected approaches over linear and non-linear signal analysis techniques without surrogate correction regarding the localization of the SOZ. These studies (33, 48, 51, 54–57, 59, 64), were based on classical intracranial EEG macro contacts, and an open question is whether a lateralization of the SOZ can also be achieved by analysing EEG recorded at the micro-scale. To address this question, we retrospectively analyzed long-term EEG recordings from epilepsy patients performed with hybrid electrodes equipped with conventional macro contacts and micro wires. In particular, we studied long-term recordings performed during nights for which a polysomnography was used to classify the different stages of the sleep-wake cycle and during which the patients had no seizures.

## 2. Materials and Methods

### 2.1. Signals

#### 2.1.1. Electroencephalographic Recordings (EEG)—Recording Techniques and Clinical Data

**Figure 1:**
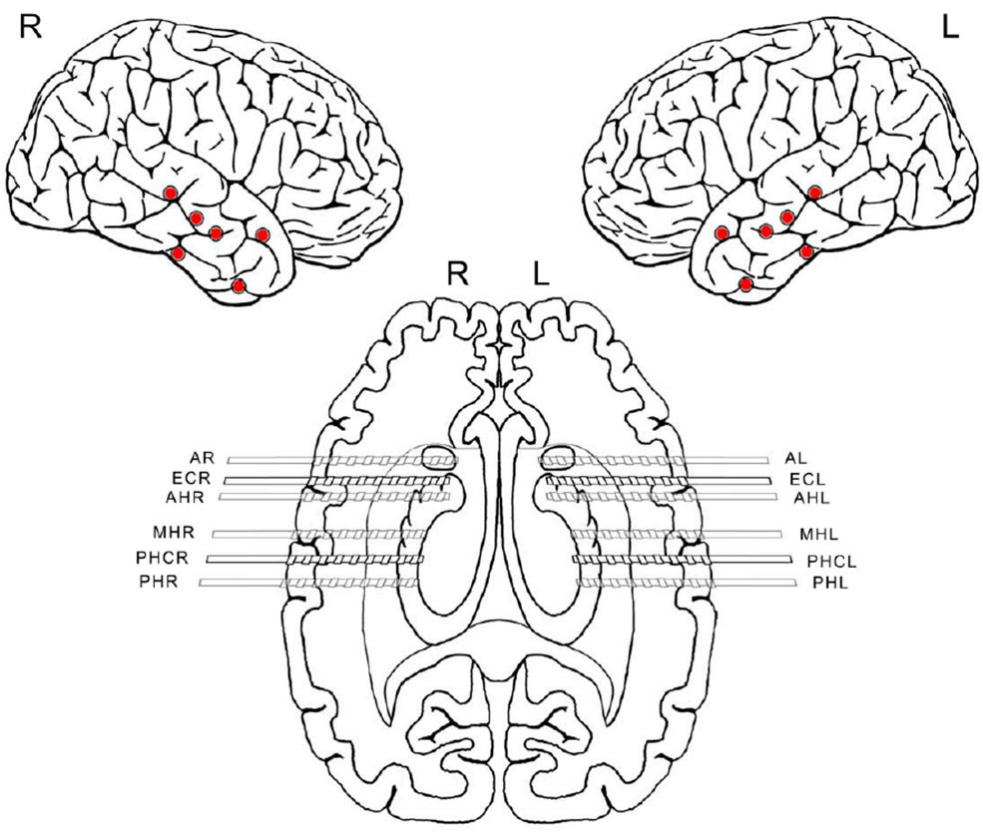
Scheme of implanted electrodes from one patient undergoing invasive pre-surgical evaluation diagnostics. Each electrode was equipped with eight macro-contacts and nine micro wires protruding from its tip (micro-wires are not displayed in the scheme). Red dots indicate the location of each hybrid depth electrode insertion. After implantation, correct electrode placement was verified using magnetic resonance imaging. The first letters indicate the brain region where the electrodes were implanted. A, amygdala; EC, entorhinal cortex; AH, anterior hippocampus; MH, middle hippocampus; PHC, parahipocampal cortex; PH, posterior hippocampus. The last letters R and L refer to the right and left hemisphere, respectively.

**Figure 2:**
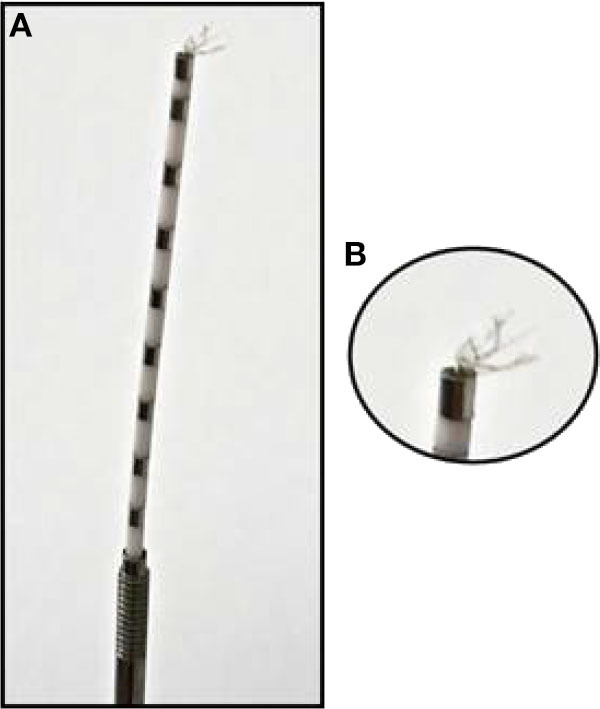
Hybrid depth electrode: **(A)** Depth electrode with eight clinical macro contacts: metal segments across the electrode spaced through white segments. **(B)** Zoom into the bundle of nine micro wires at the tip of the depth electrodes.

We analyzed a total of five intracranial EEG night recordings from three patients suffering from pharmacoresistant epilepsy. Two night recordings from two non-consecutive nights from patient A, two night recordings from consecutive nights from patient B, and one night recording from patient C. These recordings were performed in the Department of Epileptology at the University of Bonn (Germany) as part of the pre-surgical epilepsy diagnostics. These invasive recordings were necessary since non-invasive diagnostics were not sufficient to unequivocally localize the SOZ in these patients. The patients were bilaterally implanted with intracranial hybrid electrodes (AdTech, Racine, Wisconsin, USA). Implantation schemes were tailored to each individual patient, and electrode locations were defined exclusively by clinical criteria. After pre-surgical epilepsy diagnostics these patients were confirmed as having unilateral temporal lobe epilepsy (one left, two right). The total number of hybrid electrodes implanted for each patient is summarized in Table 1. Figure 1 depicts one exemplary scheme of the implanted electrodes for patients undergoing invasive pre-surgical evaluation diagnostics. The electrodes consisted of two types of contacts which recorded electrical activity of the brain at two different spatial scales. Each electrode was equipped with eight cylindrical macro contacts and contained a bundle of nine micro wires radially spaced protruding ~4 mm from its tip (Figure 2). Each bundle consisted of eight high-impedance recording electrodes and one low-impedance reference electrode. The micro wires penetrate the tissue in a non-regular way such that their final relative spatial position was different for every bundle. EEG signals were amplified using a 256-channel Neuralynx ATLAS system (Bozeman, Montana, USA). Micro-wires were additionally connected through headstages to pre-amplify the signals. After neurosurgical implantation, the placement of the electrodes was verified by magnetic resonance imaging. After the pre-surgical epilepsy diagnostics, all patients underwent epilepsy surgery. The patients' surgery outcome was classified according to Engel's classification of post-operative outcome (4). As inclusion criteria, we considered only patients who had a favorable post-operative outcome of Engel class 1 (free of disabling seizures) and Engel class 2 (only rare disabling seizures). Accordingly, prior to our analysis we had the information of which hemisphere contained the SOZ, and we could use this information as ground truth to validate our results under controlled conditions. Additionally, we included only EEG signals which were recorded simultaneously with macro electrodes and micro wires during nights in which no seizure took place. The recordings had an average length of 13.6 h per recording (range: 11.5–14.4 h) (see Table 1). Recordings were performed prior to and independently from our study as part of the epilepsy diagnostics in these patients. The results of this study did not have any impact on clinical decisions, which were made exclusively by clinical doctors. All patients had given written informed consent that their clinical data may be used for research purposes. The retrospective analysis of the EEG recordings was approved by the Medical Institutional Review Board in Bonn.

**Table 1:** Summary of clinical data.

**Patient**	**Sex**	**Age (y)**	**No. hybrid electrodes**	**SOZ**	**Surgery**	**Outcome**	**Length 1st recording (h)**	**Length 2nd recording (h)**
A	F	47	12	L	Left SAH	2	11.51	14.38
B	F	39	12	R	Right ATL	2	13.98	14.42
C	F	20	10	R	Right SAH	1a	11.66	–

*F, female; SOZ, Seizure onset zone; L, left; R, right; SAH, selective amygdalo-hippocampectomy; ATL, Anterior temporal lobectomy; Seizure outcome according to Engel classification (4)*.

#### 2.1.2. Polysomnography (PSG)

An all-night polysomnography (PSG) recording was performed along with the invasive EEG recording. The scoring of sleep stages was done manually in 30 s epochs according to the criteria of the American Academy of Sleep Medicine using the following additional scalp electrodes: C3, C4, Cb1, Cb2, F3, F4, Fz, O1, O2. In addition, electrooculogram (EOG), electromyogram (EMG), and electrocardiogram (ECG) were evaluated. The PSG to identify the intervals in which the patients were in the different stages of the sleep-wake cycle during the EEG recordings. These stages are: wakefulness state (W), rapid eye movement sleep (REM), light sleep (N1), and two slow-wave sleep states (N2 and N3). The number of windows included for each stage of the sleep-wake cycle per night of recording per patient (A 1st, A 2nd, B 1st, B 2nd, and C 1st) is given in Table 2.

**Table 2:** Number of analyzed windows per stage of the sleep-wake cycle.

		**Number of windows**					
**Patient**	**Recording**	**W**	**REM**	**N1**	**N2**	**N3**	**Total**
A	1st	807	457	187	571	222	2,244
	2nd	1,268	301	314	631	284	2,798
B	1st	1,292	136	274	610	142	2,454
	2nd	975	339	419	577	335	2,645
C	1st	1,019	211	166	547	322	2,265

#### 2.1.3. Preprocessing EEG Signals

**Figure 3:**
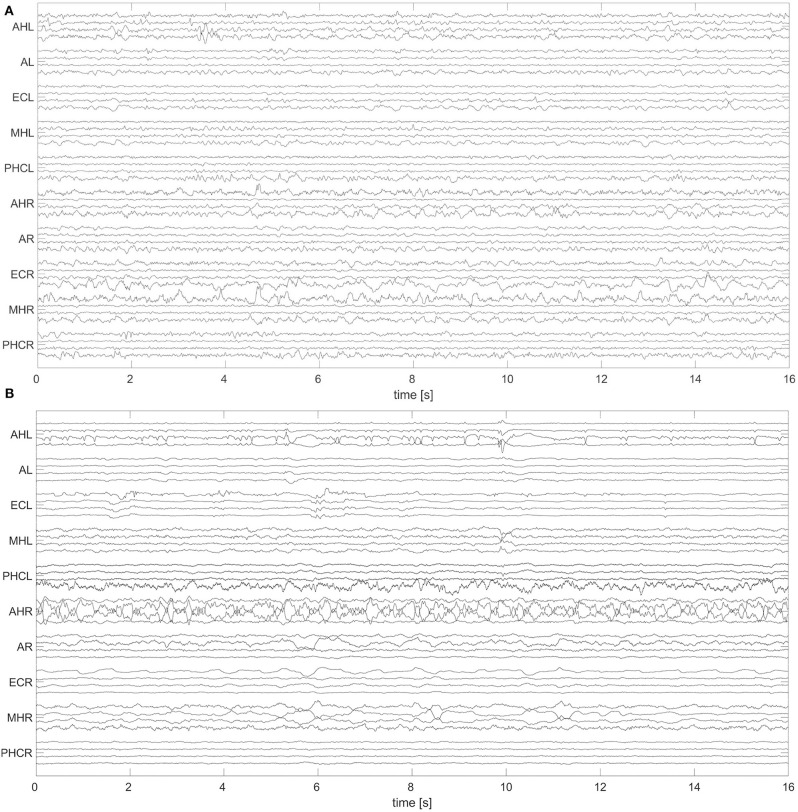
Exemplary 16 s window of an EEG recorded during the wakefulness state (W) with hybrid electrodes from patient A: The signals were recorded with macro contacts **(A)** and micro wires **(B)**. Names of the electrodes are on the vertical axes (for a further description of electrode names see caption of Figure 1). For a better visualization, in both panels we display only 40 channels to represent the 70 and 80 available macro and micro channels, respectively. For macro channels we selected four out of the seven channels per electrode selecting every second channel starting from the innermost one. For micro wires we select every second channel starting from the first channel. For this display we applied the same references and filter settings as in our analysis. For this particular patient A, the SOZ was in the left hemisphere.

**Figure 4:**
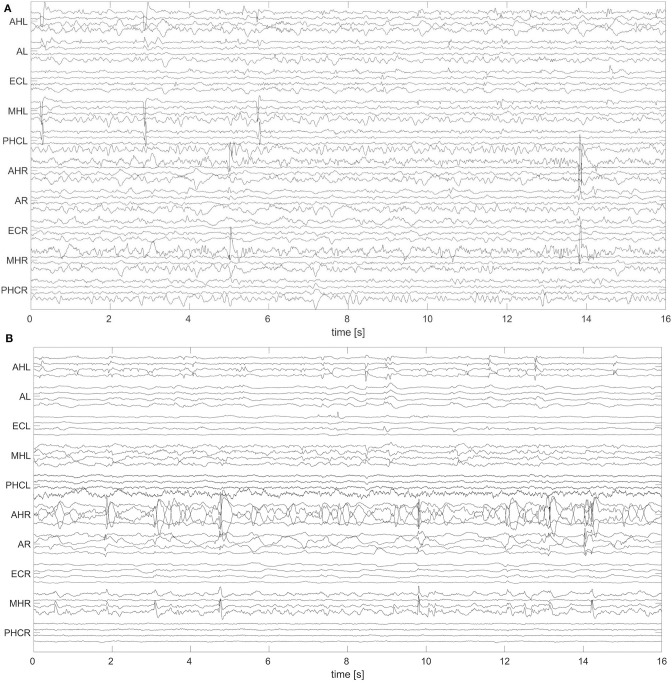
Exemplary 16 s window of an EEG recorded during deep sleep (N3) with hybrid electrodes from patient A: Same as Figure 3, but for N3.

A non-overlapping moving window of 16 s was used for the analysis of the EEG recordings. To reference macro EEG contacts we used a bipolar montage since macro contacts are equally spaced across the electrode. For bipolar montages, channels are defined from the electric potential differences of neighboring recording contacts. For micro wire bundles the spatial position of individual wires is fixed (see Figure 2). We therefore applied an electrode-wise reference. Here channels are defined by the difference between the potential at individual wires and the mean potential across the whole bundle. Subsequently, a fourth-order Butterworth band-pass filter between 0.5 and 40 Hz was used. Forward and backward filtering was applied in order to avoid phase distortion. Afterwards, signals were downsampled from 2,048 to 256 Hz. In Figure 3 we display exemplary signals from the beginning of the recording from patient A while this patient was still awake. EEG signals recorded with macro contacts are displayed in Figure 3A, whereas Figure 3B shows channels obtained from micro wire recordings. Figure 4 follows the same structure as Figure 3, but shows exemplary signals recorded during the N3 stage. In contrast to Figure 3, in Figure 4 we can see high-voltage spike-and-wave complexes, that do not correspond to physiological activity, but are characteristic for EEG recordings from epilepsy patients (100). Windows containing artifacts and channels predominantly affected by artifacts detected by visual inspection were discarded from further analysis.

### 2.2. Non-linear Signal Analysis

#### 2.2.1. Rank-Based Non-linear Predictability Score *S*

We used the rank-based non-linear predictability score (*S*) to analyse our data (the source code used in this paper for the computation of the non-linear prediction score is available at https://repositori.upf.edu/handle/10230/42940). *S* is a measure of the predictability of a system *X* based on neighboring trajectories of its dynamics (54). Assume that the scalar signal *x* was derived from the dynamical system *X* and consists of the samples *x*_*i*_ for *i* = 1, …, *N*. The first step to calculate *S* is the state space reconstruction using delay vectors with an embedding dimension *m* and time delay τ (101):

(1) $$\begin{array}{l} {\text{x}}_{i}=\left(x_{i},x_{i-\tau },\ldots ,x_{i-\left(m-1\right)\tau }\right) \end{array}$$

so that the index *i* is now restricted to *i* = η + 1, …, *N* with the embedding window η = (*m* − 1)τ. In the next step, we calculate Euclidean distances between all pairs (*i, j* = η + 1, …, *N*) of embedding vectors:

(2) $$\begin{array}{l} v_{i,j}=\sqrt{\overset{m}{\underset{d=1}{\sum }}{\left({\text{x}}_{i,d}-{\text{x}}_{j,d}\right)}^{2}} \end{array}$$

Since subsequent steps of analysis require incrementing indices by the prediction horizon *h*, which indicates the number of steps that the rank-based non-linear predictability score predicts into the future, we have to adjust also the upper limit of the time indices, in this case to *N* − *h*. For each reference state **x**_*i*_0__(*i*_0_ = η + 1, …, *N* − *h*) the distances *v*_*i, j*_ are used to find the *k* nearest neighbors: {*j*_0,*r*_}_(*r*=1,…,*k*)_, which are the *j* indices of the *k* smallest entries in the set {*v*_*i*_0_,*j*_}_(*j*=η+1,…,*N*−*h*;|*i*_0_−*j*|>*W*)_. Temporally close states are thereby excluded from these nearest neighbors by means of a Theiler correction of window length *W* (102). For calculating *S*, we do not evaluate signal amplitudes but rather ranks in sorted lists of amplitude differences. For this purpose, we calculate the distances *u*_*i, j*_ between each pair of amplitudes *x*_*i*_ and *x*_*j*_ with *i, j* = η + 1, …, *N*:

(3) $$\begin{array}{l} {\text{u}}_{i,j}=|x_{i}-x_{j}|, \end{array}$$

Subsequently, the distances {*u*_*i*_0_,*j*_}_*j*=η+1,…,*N*;|*i*_0_−*j*|>*W*_ are sorted from the lowest to the highest forming a list of ranks *g*_*i*_0_, *j*_0__. The number of differences in this set is denoted by *M*_*i*_0__. For *W* < *i*_0_ < *N* − *W* + 1 we have *M*_*i*_0__ = *N* − 2*W* − 1. Below and above this range *M*_*i*_0__ increases linearly and reaches *M*_*i*_0__ = *N* − *W* − 1 at *i*_0_ = 1 and *i*_0_ = *N*.

To test the predictability, we determine the mean rank of the amplitude differences between *x*_*i*_0_+*h*_ and the different *x*_*j*_0, *r*_+*h*_ for *r* = 1, …, *k*,

(4) $$\begin{array}{l} R_{i_{0}}=\frac{1}{k}\overset{k}{\underset{r=1}{\sum }}g_{i_{0}+h,j_{0,r}+h}. \end{array}$$

If the signal is completely predictable, *R*_*i*_0__ reduces to the mean of the *k* lowest ranks. This lowest boundary is independent of *i*_0_ is denoted by:

(5) $$\begin{array}{l} {\text{R}}^{L}=\frac{k+1}{2} \end{array}$$

In contrast, for no predictability, *g*_*i*0+*h*,*j*_0,*r*_+*h*_ are just *k* random samples taken from a uniform distribution 1, …, *M*_*i*0_. Hence the expected value in this case is:

(6) $$\begin{array}{l} {\text{R}}_{{\text{i}}_{0}}^{U}=\frac{{\text{M}}_{i_{0}}+1}{2} \end{array}$$

Finally, the rank-based prediction score is defined as follows:

(7) $$\begin{array}{l} \text{S}=\frac{1}{\text{N}-\eta -\text{h}}\overset{\text{N-h}}{\underset{{\text{i}}_{0}=1+\eta }{\sum }}\frac{{\text{R}}_{{\text{i}}_{0}}^{U}-{\text{R}}_{{\text{i}}_{0}}}{{\text{R}}_{{\text{i}}_{0}}^{U}-{\text{R}}^{L}} \end{array}$$

High values of *S* are obtained for signals measured from predictable dynamics, with an upper bound of 1 for periodic dynamics. In contrast, for uncorrelated stochastic signals, *S* has an expected value of zero.

In order to avoid any in-sample optimization of the parameters, we used the same parameter setting like in reference (54). However, to account for the difference of the sampling frequencies in our study as opposed to the one in reference (54) we adapted those parameters that are in units of time. Thus, we used *m* = 8, τ = 8 sampling times, *k* = 5, *h* = 8 sampling times, and *W* = 38 sampling times.

#### 2.2.2. Surrogate Signals

Signals measured from noise-free non-linear deterministic dynamics are predictable and therefore lead to high values of the non-linear predictability score *S*. Accordingly, *S* is sensitive to deterministic dynamics. On the other hand, the autocorrelation of signals measured from linear stochastic dynamics are also a source of predictability reflected in high *S* values. Therefore, while being sensitive, *S* is not specific for non-linear deterministic dynamics (54). This lack of specificity is not a peculiarity of the measure *S*, but affects many measures from non-linear signal analysis. This problem can be addressed by the concept of surrogates (103, 104), which allows us to test different null hypotheses about the dynamics underlying some measured signal. The particular surrogates used in this study, commonly referred to as iterative amplitude adjusted surrogates (103), represent the null hypothesis that the dynamics is a stationary linear stochastic correlated Gaussian process recorded with an invertible but potentially non-linear measurement function (103, 104). They are generated by a constrained randomization of the original signals. The constraints are such that the surrogates have the same amplitude distribution like the original signals, and the surrogates' periodogram is practically indistinguishable from the one of the original signals (103).

#### 2.2.3. Surrogate Corrected Non-linear Predictability Score ψ

We generated one surrogate signal from each signal corresponding to a window of 16 s of an individual channel. Subsequently, we computed the non-linear predictability score *S* for each signal (*S*_O_) and its surrogate (*S*_S_) to determine the surrogate corrected non-linear predictability score as:

(8) $$\begin{array}{l} \psi =S_{\text{O}}-S_{\text{S}}. \end{array}$$

The quantity *S*_S_ estimates the value of the non-linear predictability score which would be expected if the null hypothesis was true. Accordingly, for signals measured from dynamics that are consistent with the null hypothesis we expect *S*_O_ ≈ *S*_S_ and therefore ψ ≈ 0. In contrast, for non-linear deterministic dynamics *S*_O_ > *S*_S_ and ψ > 0 should hold.

#### 2.2.4. Averaging and Statistical Analysis

Once we constructed the spatio-temporal profiles, we averaged the results of ψ over time and over the electrode domain. The averages over time were taken separately for the different stages of the sleep-wake cycle, namely, W, REM, N1, N2, and N3. Due to the sleep-wake cycle, windows corresponding to a certain stage are distributed into several intervals across the night. We always included all windows from all intervals, and only windows containing transitions between these stages were not included in the averages (see Table 2 for the number of windows for each sleep-wake cycle stage in all recordings). Channels predominantly affected by artifacts and windows showing artifacts across channels were also discarded. For each stage of the sleep-wake cycle, the averages over the domain of electrode contacts were made in two steps. For the macro channels we averaged for all the macro channels belonging to the same hybrid electrode. For the micro channels, we averaged across the 8 wires contained in the individual bundle. In the second step, these electrode mean values were averaged across all electrodes implanted in the same hemisphere, resulting in hemisphere mean values.

For a statistical evaluation of the results, we applied a two-way ANOVA to check for any statistical differences in the results of ψ with respect to location (Hemisphere containing the SOZ vs. hemisphere contralateral to the SOZ) and stages of the sleep-wake cycle (W, REM, N1, N2, and N3) to each individual night recording and separately for macro electrode and micro wire recordings using a significance level of α = 0.05. *Post-hoc* analysis between groups was made using a Mann-Whitney U-test with Bonferroni correction for fifty comparisons (five stages of the sleep-wake cycle times two recording modalities times five nights), thereby adjusting the significance level from α = 0.05 to α = 0.001. Accordingly, we consider the outcome of the Mann-Whitney U-test as significant if the test resulted in *p* < 0.001. The statistical analyses were performed for the values of each 16 s window averaged across all channels belonging to the same electrode separately for macro and micro electrodes.

## 3. Results

We first look at results for one night from one of the three patients, patient A. The pre-surgical epilepsy diagnostics revealed that the SOZ was located in the left medial temporal lobe. After the surgery, this patient had a favorable outcome (Engel 2, see Table 1 for clinical details). For patient A two non-consecutive night recordings were available for analysis.

**Figure 5:**
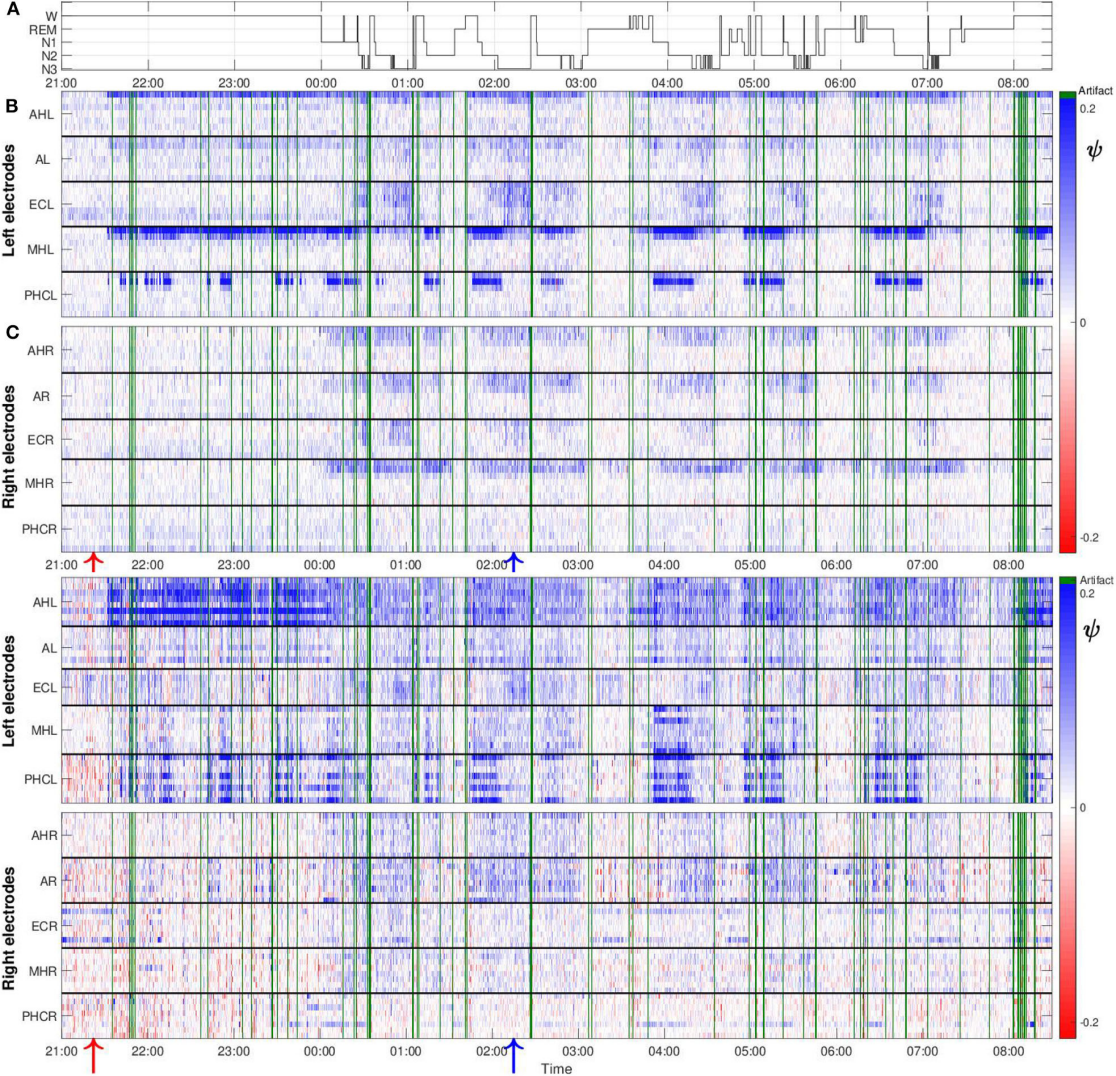
The values of ψ are higher in the hemisphere containing the SOZ as compared to the contralateral hemisphere and sleep-modulated for patient A: **(A)** Polysomnography: Display of the different stages of the sleep-wake cycle. **(B)** Color-scaled values of ψ for macro contacts. The horizontal white line separates the results from electrodes located in the left and right brain hemisphere. Horizontal black lines separate results from macro channels belonging to individual intracranial electrodes. For each electrode the inner- and outermost channel are displayed at the top and bottom, respectively. Electrode names are displayed on the vertical axes. For this particular patient, spatial gradients with regard to the extension of individual electrodes are found in both hemispheres. Higher values of ψ are found for the innermost channels, which are placed in the medial temporal lobe. These gradients are particularly strong for the MHL and PHCL electrodes. Green lines correspond to windows containing artifacts detected by visual inspection that were discarded from further analysis. One macro channel and three micro channels were excluded from the analysis and are not displayed in the profile because they predominantly contained artifacts. Red and blue arrows at the bottom, indicate the window of EEG recording displayed in Figures 3A, 4A, respectively. **(C)** Same as **(B)** but for micro wires. In contrast to macro channels, for micro channels the position with regard to black solid lines cannot indicate the spatial arrangement of the channels due to the micro electrode geometry. Therefore, spatial gradients with regard to the extension of individual electrodes cannot be tested for.

Figure 5 shows values of the surrogate corrected non-linear predictability score ψ obtained from one night recording from patient A. The polysomnography is displayed in Figure 5A. Values of ψ for the macro recordings are, in general, higher for the channels located in the hemisphere containing the SOZ as compared to the ones from the contralateral hemisphere (Figure 5B). In addition, our results do not only depend on the recording location but also on the stages of the sleep-wake cycle. In general, higher values of ψ are found for the period in which the patient was predominantly in non-REM sleep as compared to the first hours of the night in which the patient was still awake. During the REM sleep stage the values are smaller as compared to all other stages. We also obtained some negative ψ values. These can be caused by the non-stationarity of the underlying signals, making them less predictable than their surrogate.

Despite that macro and micro channels record the electrical activity of the brain at two different spatial scales, we find some similarities between the results for both recording modalities. In particular, we find that, in general, values of ψ for micro channels are higher for the hemisphere containing the SOZ (Figure 5C). Values are particularly high for the bundle of micro wires belonging to electrodes AHL and PHCL. Concerning the dependence on the sleep stages, we again find similarities between results for the macro and micro channels. Values of ψ are in general higher for light (N1) and deep sleep (N2, N3) as compared to REM sleep, and periods when the patient was awake. For this particular night, one macro channel from the electrode ECR (Figure 5B) and three micro channels (Figure 5C), two from electrode ECL and one from electrode AL, were excluded from the analysis as they were predominantly affected by artifacts. Corresponding results for the remaining four nights are displayed in the Supplementary Material. For these nights no channels had to be discarded from the analysis due to artifacts.

**Figure 6:**
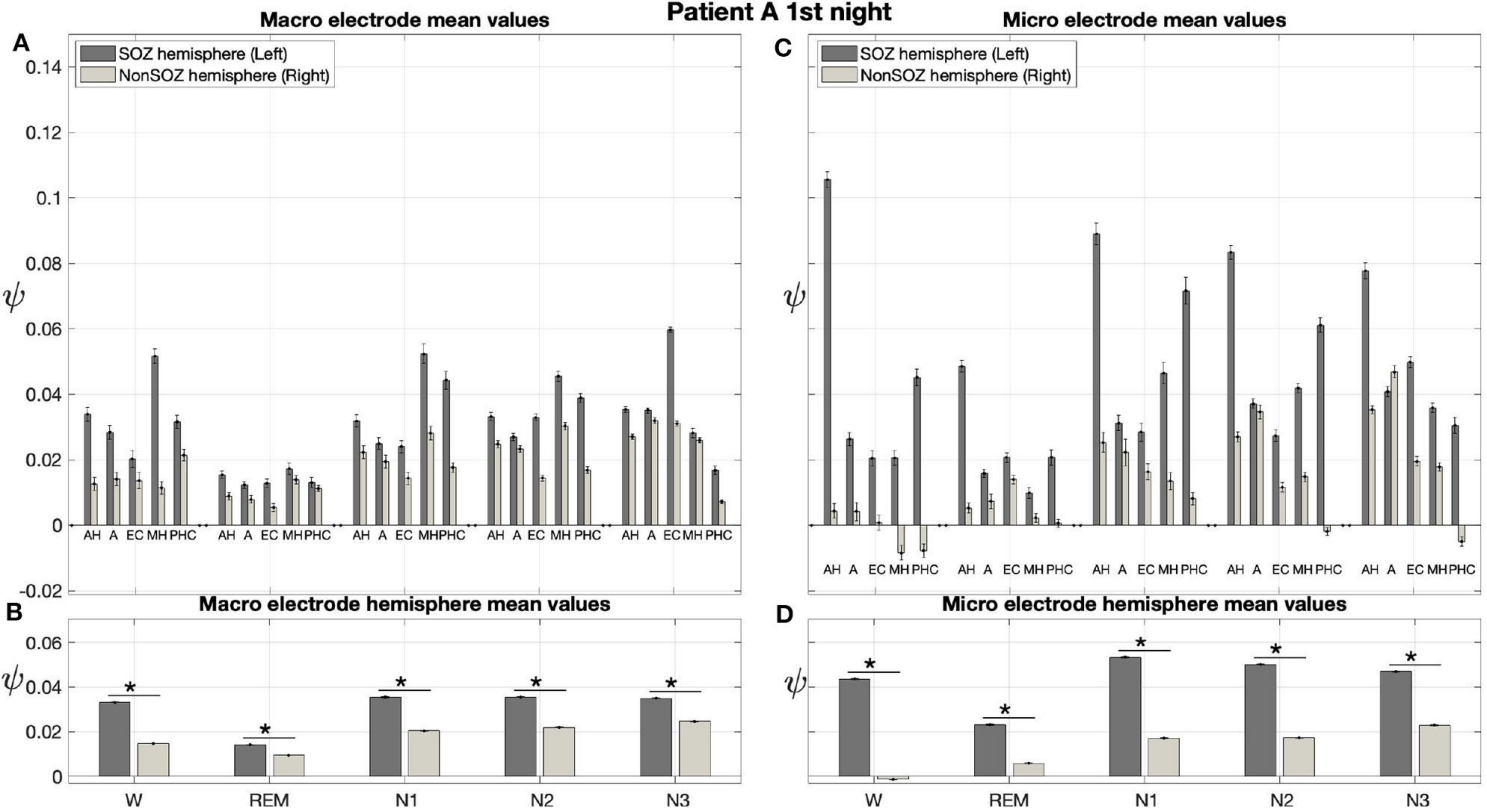
For patient A, on average ψ was higher for the hemisphere containing the SOZ (left) than for the contralateral hemisphere (right) for macro and micro EEG recordings. **(A)** Electrode mean ψ values in dependence on electrode, sleep stage, and hemisphere. Below each pair of bars, the label indicates the brain region, and the left and right bar show results for the hemisphere containing the SOZ (SOZ hemisphere) and contralateral hemisphere (Non-SOZ hemisphere), respectively. Error bars show the standard error of the mean. **(B)** Hemisphere mean ψ values in dependence on electrode, sleep stage and hemisphere. **(C,D)** Same as **(A,B)** but for micro wires. The ⋆ indicates *p* < 0.001.

The averaged results from the first night of patient A are displayed in Figure 6. In these averages, ψ was almost exclusively higher for the hemisphere containing the SOZ (left) than for the contralateral hemisphere (right) for macro and micro EEG recordings. For this night, we find a significant main effect of location and sleep stage for both macro and micro electrodes. Furthermore, there is a significant interaction between both factors with regard to ψ (Table 3).

**Table 3:** *p*-Values of the two-way ANOVA to test for statistical differences in the results of ψ with respect to location (hemisphere containing the SOZ vs. hemisphere contralateral to the SOZ) and stages of the sleep-wake cycle (W, REM, N1, N2, and N3) for macro and micro recordings.

		***p*****-values**				
**Factor technique**	**Recording**	**1st night Pat. A**	**2nd night Pat. A**	**1st night Pat. B**	**2nd night Pat. B**	**1st night Pat. C**
Location	Macro	*p* < 10^−100^	*p* < 10^−100^	*p* < 10^−10^	n.s.	*p* < 10^−100^
	Micro	*p* < 10^−100^	*p* < 10^−100^	*p* < 10^−100^	*p* < 10^−100^	n.s.
Sleep-wake	Macro	*p* < 10^−100^	*p* < 10^−100^	*p* < 10^−100^	*p* < 10^−100^	*p* < 10^−100^
cycle stage	Micro	*p* < 10^−100^	*p* < 10^−100^	*p* < 10^−100^	*p* < 10^−100^	*p* < 10^−100^
Interaction	Macro	*p* < 10^−50^	*p* < 10^−50^	*p* < 10^−10^	*p* < 10^−100^	*p* < 10^−100^
	Micro	*p* < 10^−100^	*p* < 10^−10^	*p* < 10^−50^	*p* < 10^−100^	*p* < 10^−10^

*n.s. indicates no significant difference*.

In Figure 6A the electrode mean ψ values from the macro contacts from the hemisphere containing the SOZ are always higher than the corresponding values for the contralateral hemisphere. In particular, except for N3, the MHL electrode shows the highest ψ value. The hemisphere mean values are always higher for the side containing the SOZ as compared to the contralateral side (Figure 6B).

Figures 6C,D show the results for the micro wires. Overall, these results show strong analogies to the findings we obtained for the macro electrodes. With the only exception of electrodes AL and AR for N3, we find that the electrode mean ψ values are higher for the hemisphere containing the SOZ across all stages of the sleep-wake cycle. This difference is particularly pronounced for the electrode pairs AH and PHC (Figure 6C). Higher hemisphere mean ψ values are found for all sleep stages in the side containing the SOZ as compared to the contralateral side (Figure 6D). For the micro electrodes we find that ψ values are negative not only for individual windows (see again Figure 5) but also for the electrode or even hemisphere mean values.

**Figure 7:**
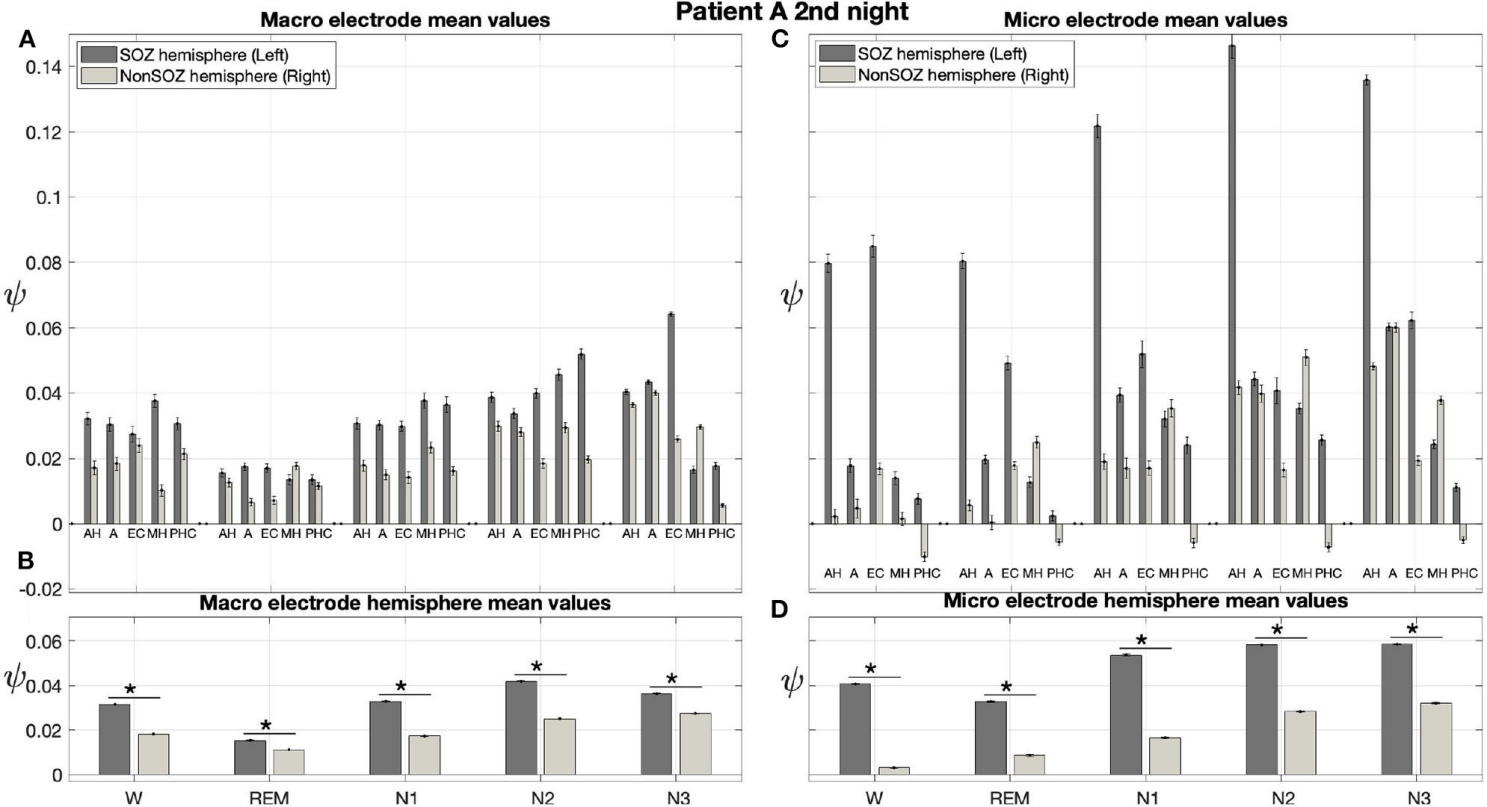
Results for ψ for patient A are consistent across nights: Same as Figure 6 but for the second night of patient A. The ⋆ indicates *p* < 0.001.

**Figure 8:**
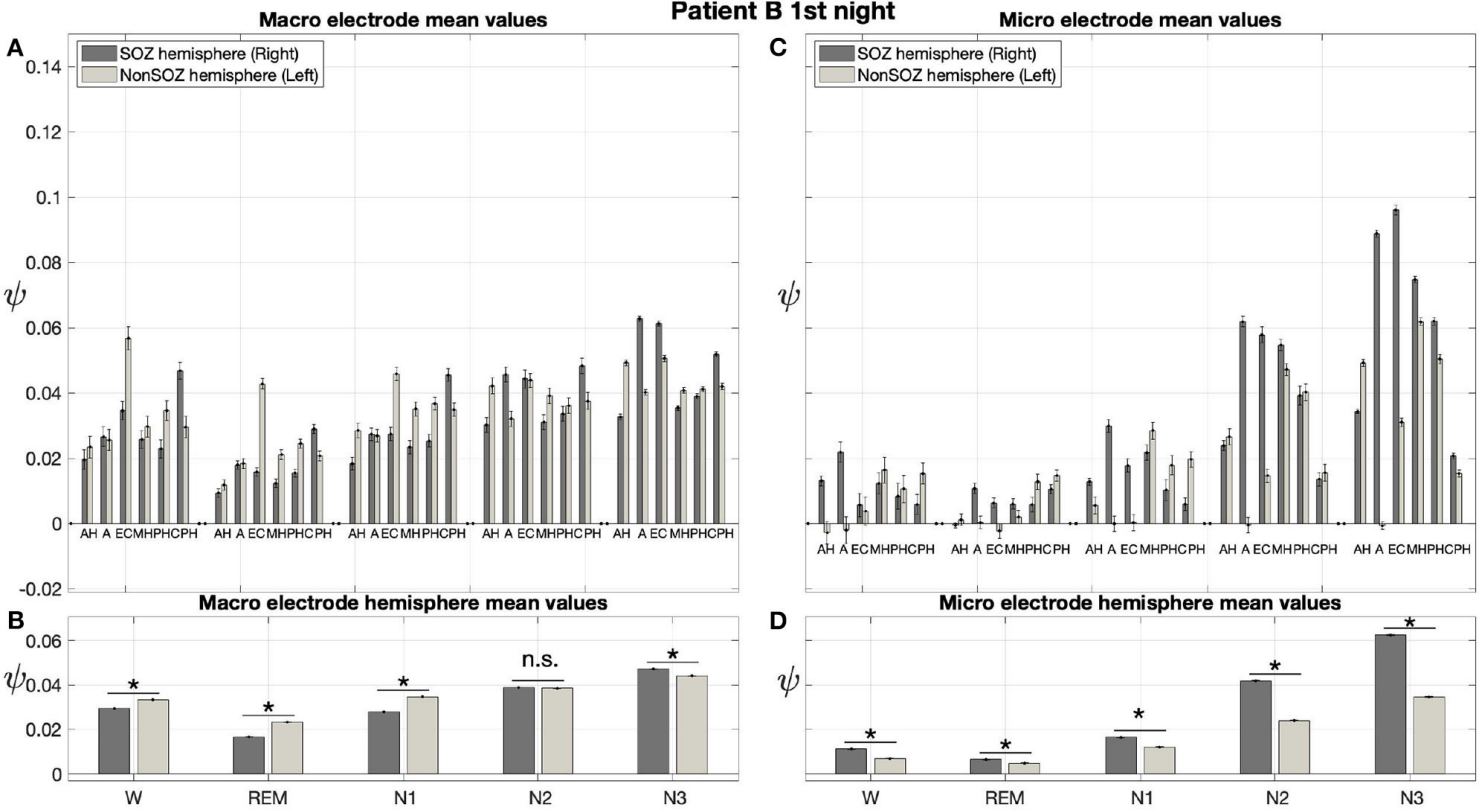
For patient B results for micro wires can help us to localize the SOZ better than the macro electrodes: Same as Figure 6 but for the first night of patient B. For patient B, the SOZ was located in the right hemisphere. The ⋆ indicates *p* < 0.001, n.s. indicates no significant difference between groups.

**Figure 9:**
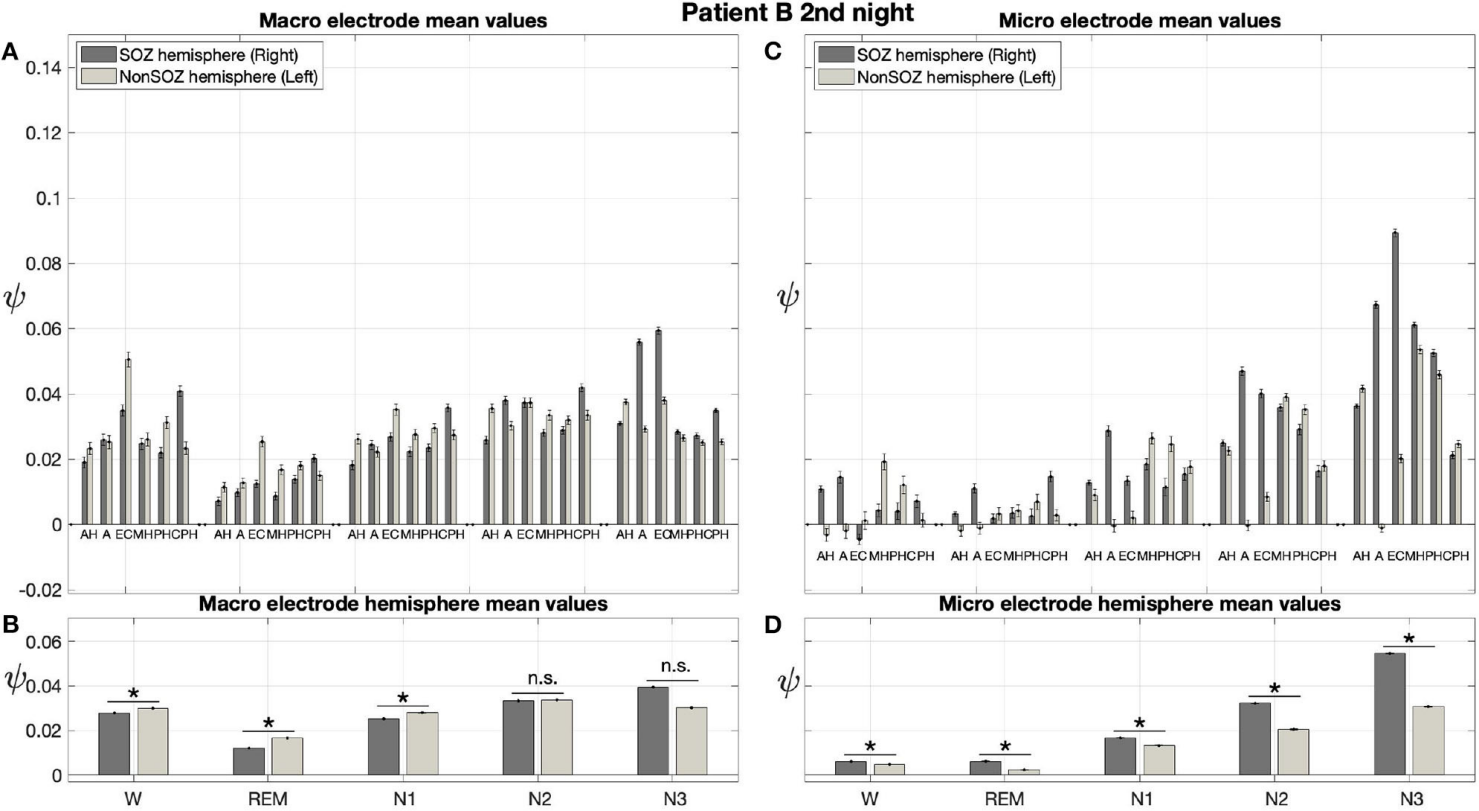
In analogy to patient A, results for patient B are consistent across nights: Same as Figure 6 but for the second night of patient B. The ⋆ indicates *p* < 0.001, n.s. indicates no significant difference between groups.

Results for a second night of patient A are shown in Figure 7. For this second night we obtain the same main findings with only some exceptions. Again we find a significant effect of location, sleep stage and its interaction on ψ for macro and micro electrode recordings (Table 3). However, for macro recordings, the electrode MHL is no longer outstanding. Furthermore, for REM and N3 this electrode shows lower values in the hemisphere containing the SOZ as compared to the contralateral hemisphere (Figure 7A). Nonetheless, once averaged to the hemisphere mean values, ψ is significantly higher in all sleep stages for the hemisphere containing the SOZ (Figure 7B). Regarding micro wires, results for electrode AHL continue to be the highest except for the wakefulness state, where instead electrode ECL shows the highest value of ψ (Figure 7C). In contrast, the electrode PHC no longer stands out from the rest. Concerning hemisphere mean values for micro electrodes, values are always higher in the hemisphere containing the SOZ as compared to those of the opposite hemisphere (Figure 7D). As a whole, Figures 6, 7 reveal an across-night consistency of the ψ value for the two nights of patient A. For patient B, the pre-surgical epilepsy diagnostics revealed that the SOZ was in the right temporal lobe. The outcome of the epilepsy surgery for this patient was favorable (Engel 2, see Table 1 for clinical details). For this patient two recordings from two consecutive nights were available. In analogy to the results obtained for both nights of patient A, for the first night of patient B, we find both a significant main effect of location, sleep stage and interaction between both factors on ψ for macro and micro electrodes (Figure 8; Table 3). In contrast to patient A, no individual macro electrode shows outstanding values of ψ, and in general smaller differences between the mean values of the hemisphere containing the SOZ and the contralateral hemisphere are found (Figures 8A,B). In fact, for W, REM sleep, and N1 we find higher values of ψ for the hemisphere that do not contained the SOZ, which is contrary to our findings from patient A. On the other hand, for N2 and N3 hemisphere mean values for the side containing the SOZ are higher than for the contralateral side which is again analogous to results for patient A. Although these differences are not significant for N2 (*p* = 0.002), for N3 this difference becomes significant (Figure 8B; Table 4). Turning to the results of the micro wires, we see that in the majority of the cases, higher electrode mean ψ values are found for the hemisphere containing the SOZ (Figure 8C). At the level of hemisphere mean values, significantly higher values of ψ are obtained for the side containing the SOZ across all sleep stages (Figure 8D). Accordingly, for the micro wires our results for the first night of patient B are consistent with the obtained for the two nights of patient A. Analysing a second night of patient B (Figure 9) we again find across-night consistency of our results (Figures 8, 9). The only differences with regard to the first night of patient B are the following. We do not get a significant effect of sleep stage (*p* = 0.567) on ψ for macro electrodes (Table 3). Furthermore, while higher ψ values for the hemisphere containing the SOZ remain not significant for N2 (*p* = 0.083), for N3 this difference becomes also not significant (*p* = 0.023) (Figure 9B; Table 4).

**Table 4:** *p*-values of the Mann-Whitney *U*-test with Bonferroni correction for fifty comparisons. n.s. indicates no significant difference.

		***p*****-values**				
**Patient and night recording**	**Recording technique**	**W**	**REM**	**N1**	**N2**	**N3**
A—1st	Macro	*p* < 10^−100^	*p* < 10^−10^	*p* < 10^−50^	*p* < 10^−100^	*p* < 10^−10^
	Micro	*p* < 10^−100^	*p* < 10^−100^	*p* < 10^−100^	*p* < 10^−100^	*p* < 10^−50^
A—2nd	Macro	*p* < 10^−100^	*p* < 10^−10^	*p* < 10^−100^	*p* < 10^−100^	*p* < 10^−10^
	Micro	*p* < 10^−100^	*p* < 10^−50^	*p* < 10^−100^	*p* < 10^−100^	*p* < 10^−10^
B—1st	Macro	*p* < 10^−3^	*p* < 10^−10^	*p* < 10^−10^	n.s.	*p* < 10^−10^
	Micro	*p* < 10^−3^	*p* < 10^−10^	*p* < 10^−10^	*p* < 10^−50^	*p* < 10^−50^
B—2nd	Macro	*p* < 10^−10^	*p* < 10^−10^	*p* < 10^−10^	n.s.	n.s.
	Micro	*p* < 10^−10^	*p* < 10^−3^	*p* < 10^−3^	*p* < 10^−50^	*p* < 10^−10^
C—1st	Macro	*p* < 10^−100^	*p* < 10^−50^	*p* < 10^−10^	*p* < 10^−10^	*p* < 10^−100^
	Micro	*p* < 10^−10^	*p* < 10^−10^	n.s.	*p* < 10^−10^	*p* < 10^−10^

**Figure 10:**
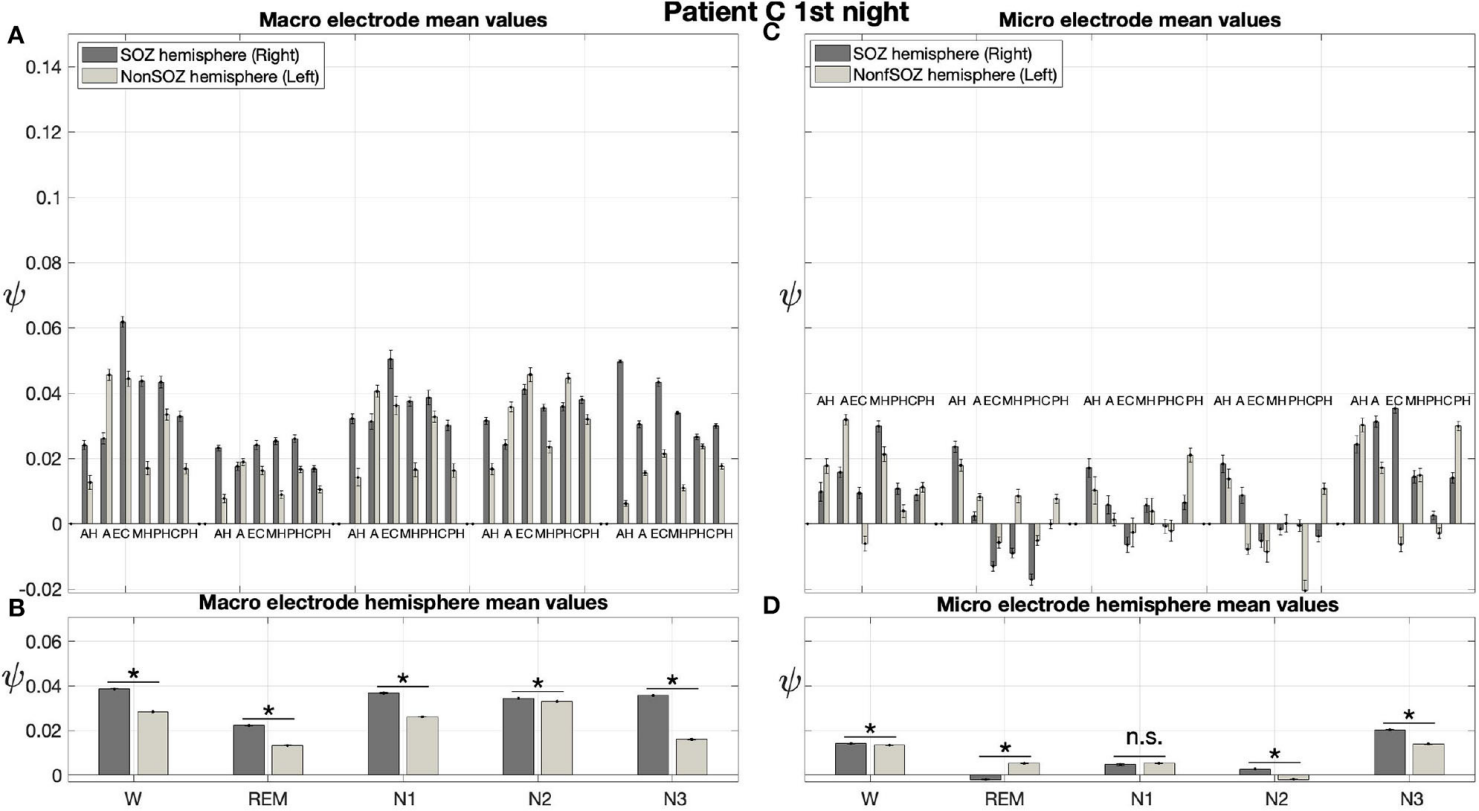
For patient C results for macro electrodes can help us to localize the SOZ better than the micro wires: Same structure as Figure 6 but for patient C. For patient C, the SOZ was located in the right hemisphere. The ⋆ indicates *p* < 0.001, n.s. indicates no significant difference between groups.

Pre-surgical epilepsy diagnostics for patient C showed that the SOZ was in the right medial temporal lobe. After the epilepsy surgery, patient C had a favorable outcome (Engel 1a, see Table 1 for clinical details). For this patient only a recording of one night was available. For this recording we obtain no significant effect of sleep stage on ψ (*p* = 0.144) for micro electrodes. In contrast, in analogy to our findings for patient A and patient B, we find a significant main effect of location, and an interaction between location and sleep stage (Figure 10; Table 3). For the micro electrode mean values, there is a substantial variability across stages of the sleep-wake cycle and different electrodes, and no systematic difference between values of the hemisphere containing the SOZ and the contralateral hemisphere can be discerned (Figure 10C). Nevertheless, for W, N2, and N3 the hemisphere mean values are significantly higher in the hemisphere containing the SOZ as compared to the contralateral hemisphere (Figure 10D; Table 4). Concerning macro electrodes, a significant main effect of location, sleep stage and the interaction between both factors is found (Table 3). In the majority, electrode mean values for the hemisphere containing the SOZ are higher as compared to the contralateral electrodes. The exceptions are found among the results for N2 and electrode A (Figure 10A). The hemisphere mean ψ values for the macro electrodes in the side containing the SOZ are significantly higher than the ones for the contralateral side across all sleep stages (Figure 10B). This finding of higher values for the hemisphere containing the seizure onset zone is again in good agreement to the findings for both nights of patient A. In order to test for consistencies in a quantitative way across nights and patients, more intracranial EEG night recordings would be needed.

When we pool the results across all three patients and five nights, we have 140 comparisons between the hemisphere containing the SOZ and the contralateral hemisphere on the level of electrodes (five stages of the sleep-wake cycle times five electrodes times two nights of patient A and five stages of the sleep-wake cycle times six electrodes times two and one nights of patient B and C, respectively). In this study we do not aim at a precise localization of the SOZ, but for a lateralization of the hemisphere containing the SOZ. Higher values for the electrodes in the hemisphere containing the SOZ are found in 95 and 93 comparisons for macro electrodes and micro wires, respectively. This corresponds to 67.9 and 66.4%, respectively. This is higher than the chance level of 50%, which would be obtained by randomly selecting one of the two hemispheres as the one containing the SOZ. Under the assumption that the different comparisons are independent, the probability of obtaining these or even higher percentages by chance are *p* = 1.4 × 10^−5^ for macro and *p* = 6.3 × 10^−5^ for micro electrode mean values. At the level of hemisphere mean values, we have 25 comparisons (five stages of the sleep-wake cycle times five nights). We get 19 (76.0%) and 23 (92.0%) times higher values for the hemisphere containing the SOZ for macro electrodes and micro wires, respectively. The probabilities of obtaining these or even higher percentages by chance are *p* = 7.3 × 10^−3^ and *p* = 9.7 × 10^−6^ for macro and micro hemisphere mean values, respectively. In particular, for sleep stages N2 and N3 the hemisphere mean values are always higher for the side containing the SOZ for both recording modalities.

## 4. Discussion

In this study, we applied a non-linear signal analysis technique to long-term intracranial EEG recordings from epilepsy patients. The recordings were performed with hybrid depth electrodes which are composed by a combination of macro contacts and micro wires. It extends previous studies that characterized the seizure-free interval, which were exclusively based on macro contacts (23–44, 46–62). While some of these studies based only on macro contacts include big samples sizes [e.g., (34–36, 42, 56)], studies based on both macro contacts and micro wires typically include a lower number of patients (74, 76, 80, 81, 97) because of the limited number of patients in which these combined recordings are performed. Leading beyond this state of the art, our study represents the first application of non-linear signal analysis to such type of EEG recordings.

As non-linear signal analysis technique we used the surrogate corrected non-linear predictability score (ψ). This method aims at discriminating non-linear deterministic dynamics from linear stochastic dynamics. The main result of our study is that the mean values of ψ are, in their majority, higher when calculated for the EEG recorded in the hemisphere containing the SOZ as compared to the contralateral hemisphere.

While we use the surrogate correction as a baseline correction for the non-linear predictability score, it represents at the same time the testing of the following null hypothesis. The dynamics are a stationary linear stochastic correlated Gaussian process recorded with an invertible but potentially non-linear measurement function (48, 104). The higher ψ values found for the hemisphere containing the SOZ imply that the underlying neuronal dynamics are less consistent with this null hypothesis as compared to the dynamics of the contralateral hemisphere. This result which we obtain from both macro and micro EEG recordings is consistent with previous studies which were based on intracranial macro EEG recordings and a variety of signal analysis techniques (11, 14, 33, 48, 51, 54–57, 59, 64). It is important to point out that the surrogates' null hypothesis is comprised by several assumptions. If any of these assumptions is not fulfilled, the null hypothesis should be rejected. Accordingly, such a rejection does not prove that our signals are recorded from a deterministic dynamical system. Alternative interpretations include that the underlying dynamics are non-stationary, or non-Gaussian, or that the measurement function is not invertible. Keeping these limitations in mind, we conclude that the epileptic process induces or enhances non-linear deterministic structures in EEG recordings (9, 11, 48, 54, 56, 64).

Our results show prominent variability with regard to the recording modalities and across patients. For patient A, increased ψ values for the hemisphere containing the SOZ were obtained for both macro and micro EEG recordings across all stages of the sleep-wake cycle. For patient B, this was found only for the micro EEG recordings. In contrast, for this patient's macro EEG recordings, increased ψ values for the hemisphere contralateral to the SOZ or non-significant differences were found. For the macro EEG recordings of patient C, we found increased ψ values for the hemisphere containing the SOZ across all stages of the sleep-wake cycle. For the micro EEG recordings this was found for wakefulness, N2 and N3 only. As we will discuss in more detail below, there are various factors that can contribute to these differences in the results across patients. These include interictal epileptiform activity (51, 105), levels of medications (106), or proximity of the various electrodes to the exact site of the seizure onset zone in the individual patients.

When we pooled the results across all three patients and five nights, we found that the values of ψ were higher for the hemisphere containing the SOZ, as compared to the contralateral hemisphere for 95 and 93 comparisons for macro and micro electrodes out of 140, respectively. This represents a correct lateralization of the SOZ for the 67.9% (95 of 140) of the macro electrodes and a 66.4% (93 of 140) of the micro wires. In contrast, when we pooled at the level of hemisphere mean values, the accuracy regarding the lateralization of the SOZ using micro wire recordings (92.0%) (23 of 25) was better as compared to the accuracy using macro contacts (76.0%) (19 of 25). This combined analysis of macro contacts and micro wires may therefore help to further improve the degree to which quantitative EEG analysis can contribute to the diagnostics in epilepsy patients.

Regarding the stages of the sleep-wake cycle, N1 and N2 are known to increase the generalized spike-wave discharges and N3 activates mainly interictal spikes (78, 105, 107, 108). On the other hand, waking state and REM sleep inhibit interictal activity (105). Previous studies that used non-linear signal analysis measures in combinations with surrogates (51, 57, 59, 64) showed that more prominent indications of non-linear deterministic structures can be caused by interictal epileptiform activity. Our measure is neither completely independent from this type of activity, nor fully determined by it. Prominent interictal epileptiform activity will likely be picked up by our technique, but also more subtle characteristics that may go unnoticed by visual inspection as confirmed by a pre-analysis of exemplary data. It can therefore be conjectured that the modulation of ψ values reflects the variability of interictal epileptiform activity across different stages of the sleep-wake cycle. Furthermore, this variability can be explained by factors, such as the level of anti-seizure medications (106). Additionally, one should note that invasive EEG recordings are nowadays used only for more complicated cases as compared to the patient groups from earlier studies. For this reason, a lateralization of the SOZ in these patients by means of quantitative EEG analysis is also more challenging. The number of night recordings included in this study is limited and further studies on bigger sample sizes will be needed to further substantiate our conclusions. Furthermore, a precise localization of the SOZ with regard to individual macro contacts, instead of only a lateralization, should be addressed. These aspects will be important to better assess the utility of such an analysis to contribute to the presurgical diagnosis in epilepsy patients.

## Data Availability Statement

The original contributions presented in the study are included in the article/Supplementary Material, further inquiries can be directed to the corresponding author/s.

## Ethics Statement

The studies involving human participants were reviewed and approved by Medical Institutional Review Board in Bonn. The patients/participants provided their written informed consent to participate in this study. Written informed consent was obtained from the individual(s) for the publication of any potentially identifiable images or data included in this article.

## Author Contributions

CM and RA devised the analysis of the EEG recordings, discussed the results, and wrote the paper. CM carried out the numerical data analysis. FM and JN carried out the clinical evaluation of the EEG recordings. All authors contributed to the article and approved the submitted version.

## Conflict of Interest

The authors declare that the research was conducted in the absence of any commercial or financial relationships that could be construed as a potential conflict of interest.
