# Supplementary material for: Seizure Onset Zone Lateralization Using a Non-linear Analysis of Micro vs. Macro Electroencephalographic Recordings During Seizure-Free Stages of the Sleep-Wake Cycle From Epilepsy Patients
Source: Front Neurol. 2020 Sep 17;11:553885. doi: 10.3389/fneur.2020.553885 (PMC7527464; doi:10.3389/fneur.2020.553885)
Supplement: Supplementary file 1 [file Data_Sheet_1.PDF]

Supplementary Material

1 SUPPLEMENTARY FIGURES

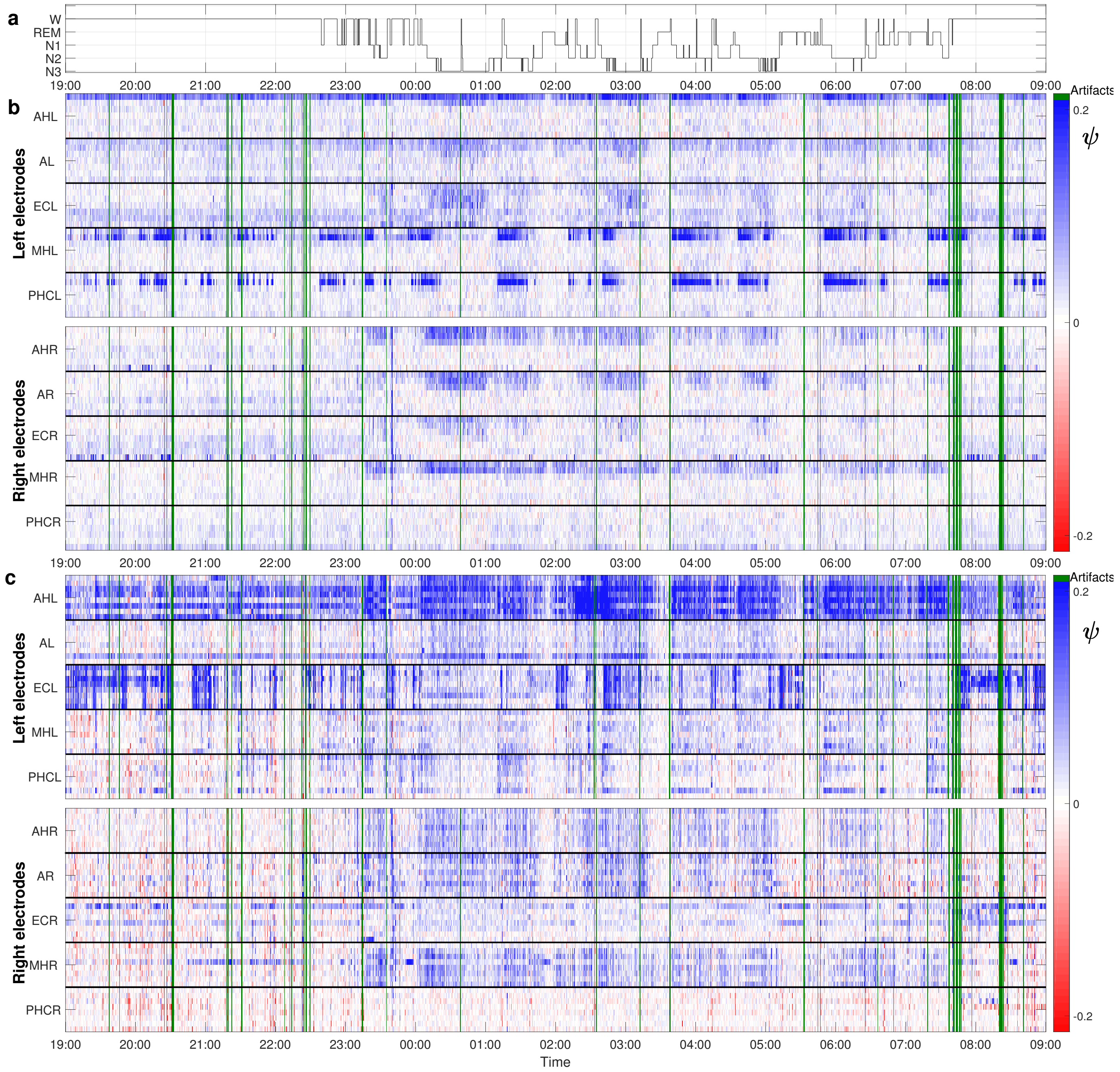

Figure S1. The values of ψ are higher in the focal hemisphere as compared to the nonfocal hemisphere and sleep-modulated for the second night of patient A: **(A)** Polysomnography: Display of the different stages of the sleep-wake cycle. **(B)** Color-scaled values of ψ for macro contacts. The horizontal white line separates the results from electrodes located in the left and right brain hemisphere. Horizontal black lines separate results from macro channels belonging to individual intracranial electrodes. For each electrode the inner- and outermost channel are displayed at the top and bottom, respectively. Electrode names are displayed on the vertical axes. Green lines correspond to windows containing artifacts detected by visual inspection that were discarded from further analysis. One macro channel and three micro channels were excluded from the analysis and are not displayed in the profile because they predominantly contained artifacts. **(C)** Same as panel **D)** but for micro wires. In contrast to macro channels, for micro channels the position with regard to black solid lines cannot indicate the spatial arrangement of the channels due to the micro electrode geometry.

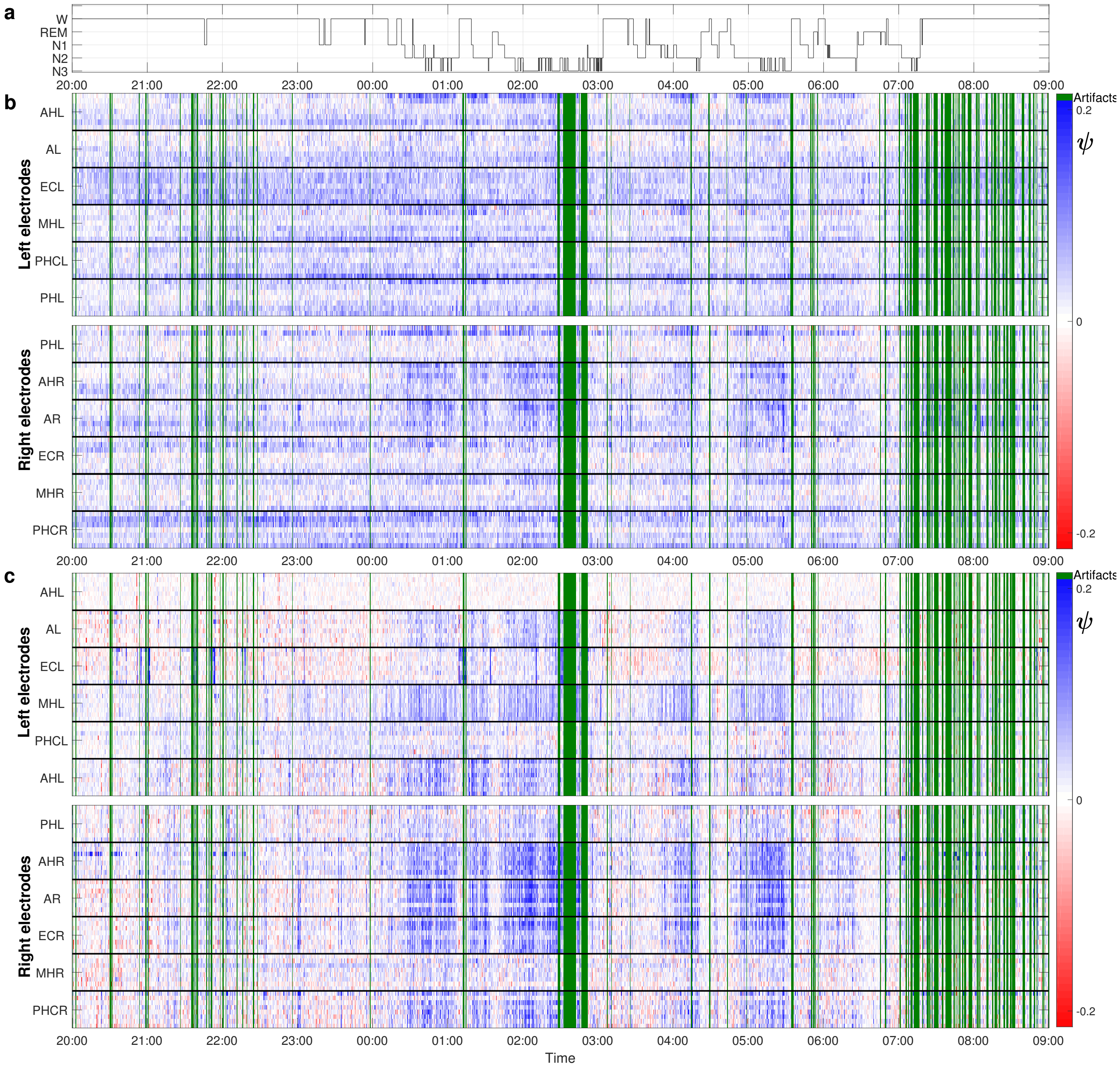

Figure S2. Results of ψ for the first night of recordings for patient B: Same as Suppl. Fig. S1

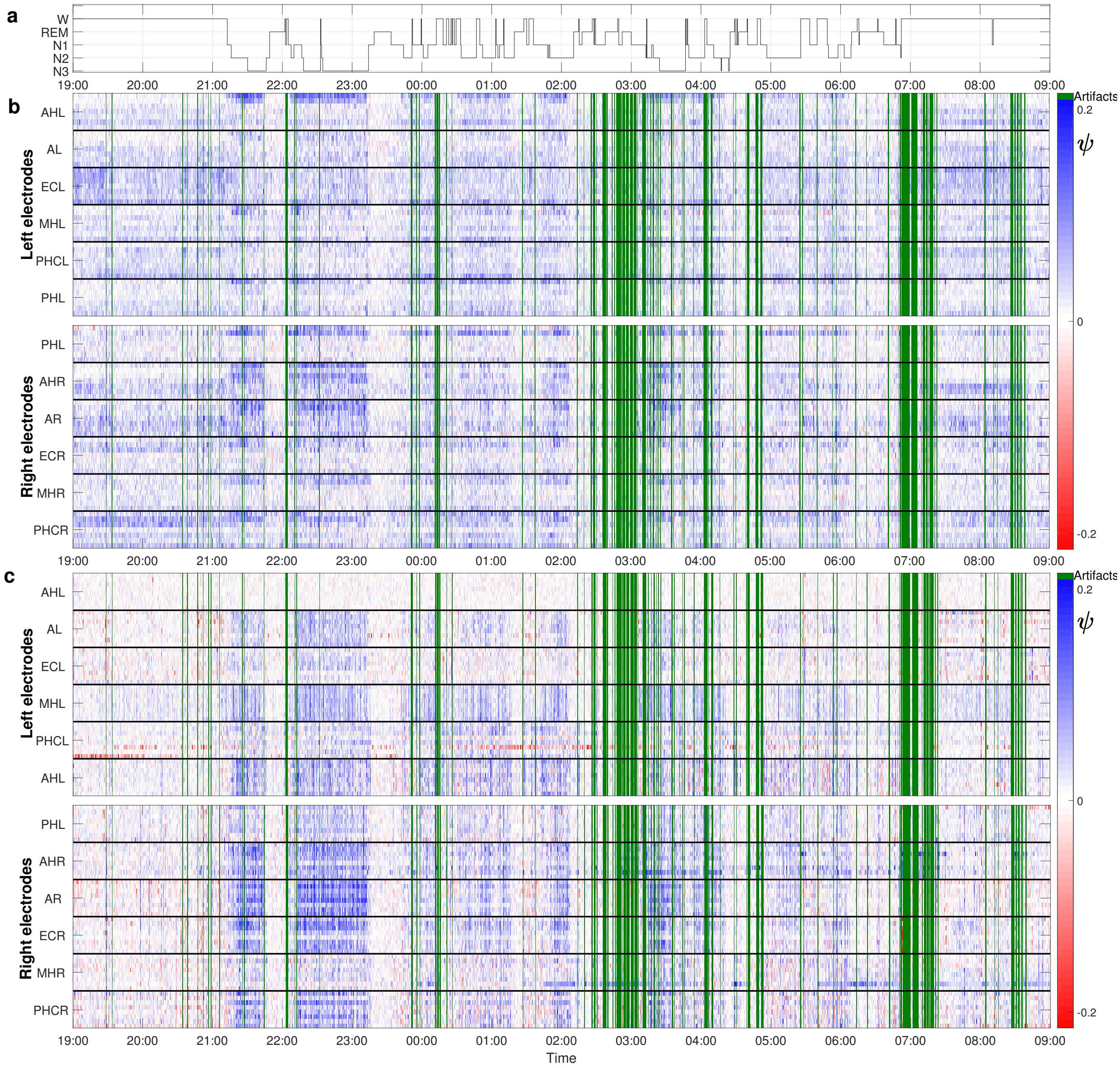

Figure S3. Results of ψ for the second night of recordings for patient B: Same as Suppl. Fig. S1

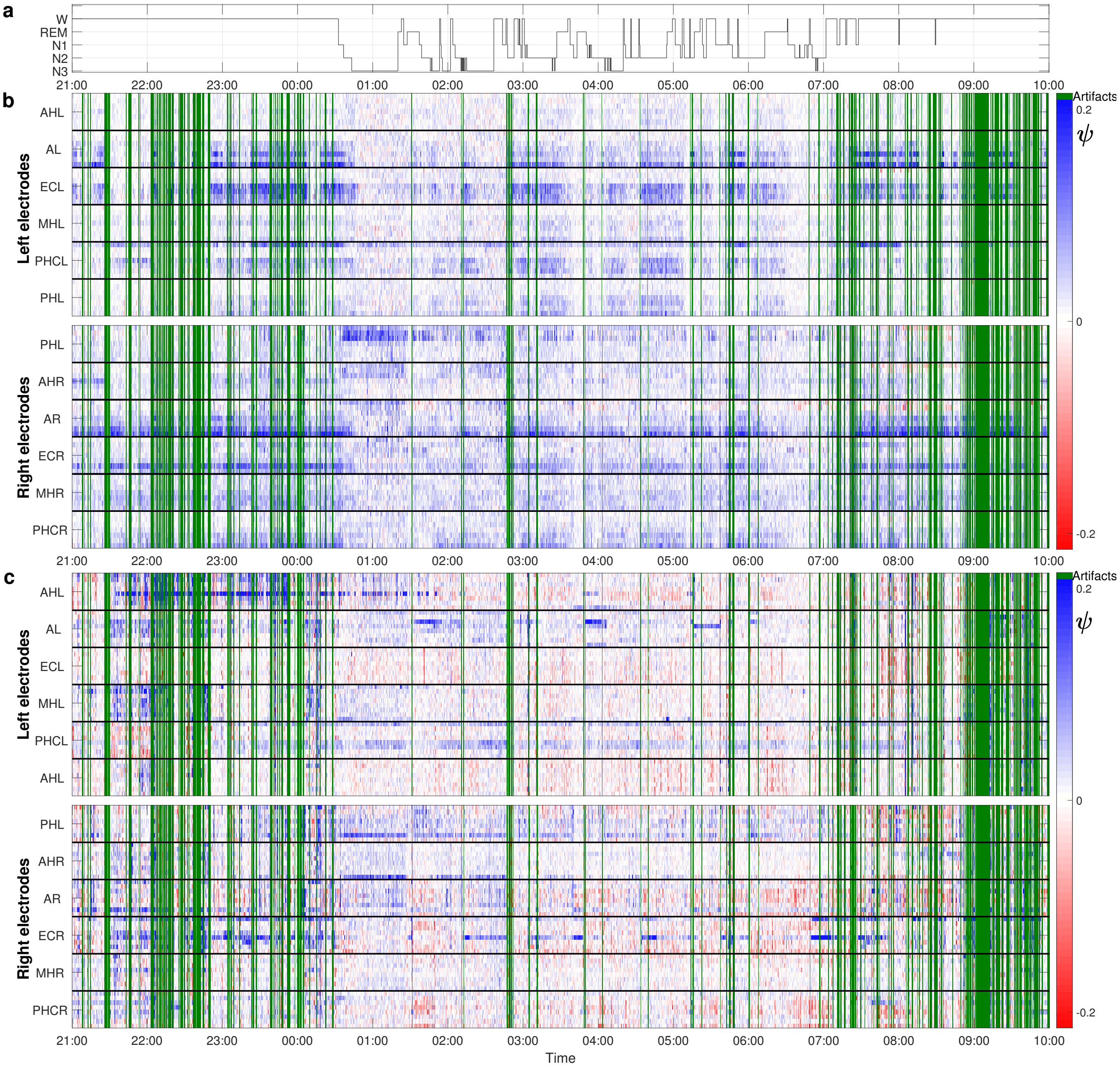

Figure S4. Results of ψ for the first night of recordings for patient C: Same as Suppl. Fig. S1
